# Impact of Obesity on In Vitro Fertilization (IVF) Outcomes: A Systematic Review

**DOI:** 10.7759/cureus.90695

**Published:** 2025-08-21

**Authors:** Sruthi Boddeti, Khutaija Noor, Mehwish Ansar, Mahlet Abera, Suchith B Suresh, Aparna Malireddi, Safeera Khan

**Affiliations:** 1 Medicine, California Institute of Behavioral Neurosciences & Psychology, Fairfield, USA; 2 Obstetrics and Gynaecology, Cleveland Clinic Florida, Weston, USA; 3 Foundations of Clinical Research, Harvard Medical School, Boston, USA; 4 Neuropsychiatry, PsychCare Consultants Research, Saint Louis, USA; 5 Internal Medicine, Shadan Institute of Medical Sciences, Peeramcheru, IND; 6 General Surgery, Wirral University Teaching Hospital, Wirral, GBR; 7 Internal Medicine, California Institute of Behavioral Neurosciences & Psychology, Fairfield, USA; 8 Internal Medicine, Montefiore St. Luke's Cornwall, Newburgh, USA; 9 Internal Medicine, Andhra Medical College, Visakhapatnam, IND

**Keywords:** assisted reproductive techniques (art), infertility treatment, in vitro fertilization, ivf outcomes, obesity, pregnancy rates

## Abstract

Obesity is a prominent health issue across the globe that causes negative reproductive consequences like decreased fertility. Even its effects on the success of in vitro fertilization (IVF) have drawn interest, with some studies indicating that BMI was linked to IVF failure. In this systematic review, we attempt to bring together the data regarding the impact of obesity on IVF outcomes. It also intends to quantify the adverse effects of female partner obesity on key IVF measures such as implantation, clinical pregnancy rate (CPR), live birth rate (LBR), and miscarriage rate (MR), and to understand the mechanism of these adverse effects in obese women.

A literature search was undertaken in PubMed/Medline, PubMed Central, Cochrane Library, Science Direct, and MDPI for studies comparing the effects of obesity (BMI ≥ 30 kg/m²) on IVF results. These studies included randomized controlled trials, cohort studies, case studies, literature reviews, and case-control studies. From the databases, we were able to select 533 relevant articles. The studies were evaluated, eligibility standards were followed, and 13 papers were considered. We collected data, and analyses of implantation rates, CPR, LBR, and MR were narratively reviewed because the studies were heterogeneous. This review was conducted using the Preferred Reporting Items for Systematic Reviews and Meta-Analyses (PRISMA) guidelines, and the quality was assessed using the Cochrane Risk of Bias 2 scale for randomized trials and the Newcastle-Ottawa Scale for observational studies.

A total of 13 studies, including participants, were included in the analysis. Obesity was consistently correlated with lower implantation, reduced CPR and LBR, and higher MR than women with normal BMI. To be precise, every five-unit increase in BMI was associated with a 5-7% reduction in CPR and LBR and a 9% rise in MR, underscoring the significant impact of obesity on IVF outcomes. In terms of mechanisms, metabolic disturbances in the oocytes, reduced endometrial response, and chronic inflammation were identified as the primary causes of these poor outcomes in obese women. In addition, interventions to reduce weight, such as diet and bariatric surgery, had an impact on the success of IVF.

Obesity has a significant negative effect on IVF success, both in terms of oocyte quality and endometrial responsiveness. In overweight women who are seeking fertility treatment, weight control must be part of preconception treatment. However, further studies are needed to determine effective weight loss interventions and to better understand the mechanisms underlying IVF success in this group.

## Introduction and background

Obesity is a global public health crisis that is growing at an alarming rate. Worldwide, the obese adult population stands at over 650 million, with women representing a significant portion of this population. Obesity has been shown to be linked to reproductive issues, such as poor oocyte quality, anovulation, menstrual irregularities, and infertility. The research suggests that women with a BMI of 30 kg/m^2^ or more will be at increased risk for infertility in nature and via assisted reproductive technology (ART), like in vitro fertilization (IVF) [1-5].

The impact of body fat distribution on fertility was also well researched by Zaadstra et al., where they concluded that, with each 0.1 unit increase in waist-hip ratio, the probability of conception per cycle was 30% lower (hazard ratio: 0.706; 95% confidence interval: 0.562 to 0.887) after adjusting for age, BMI, the reason for artificial insemination, cycle length and regularity, smoking, and parity [6].

One of the most common ART procedures is IVF, which involves stimulation of the ovaries, egg retrieval, lab fertilization, and then embryo transfer into the womb [2]. IVF has changed the world of reproductive medicine, yet clinical pregnancy rates (CPRs) and live birth rates (LBRs) are lower in obese IVF patients [3]. A dose-response relation between rising BMI and lower IVF success has been observed [7]. Also, obese women have more miscarriages, which must be because of metabolic and endocrine imbalances within the maternal uterine environment.

Significance of IVF

This worldwide infertility problem, with 10-15% of couples at risk, makes IVF all the more critical. The procedure now has greater application to a variety of new age scenarios, from same-sex partners to divorced mothers and fathers to people seeking fertility preservation (such as cancer patients). The social importance of IVF is not only for infertility but also includes preimplantation genetic testing (PGT) for detecting genetic diseases [8].

The human and emotional aspects of IVF

IVF is not just a medical procedure; it is a deeply personal experience with significant financial, physical, and emotional stakes. It is a rigorous process, with repeating cycles, hormone injections, and clinic visits. Every step of the process, from egg retrieval to embryo transfer, is fraught with anxiety and anticipation, and many people have to live with uncertainty. These emotional difficulties are augmented by the stress of potentially lost cycles [9].

Rationale

Despite the known associations between obesity and poorer reproductive outcomes, the precise mechanisms by which obesity affects IVF success remain under investigation. Obesity-related factors, such as altered follicular fluid composition, impaired oocyte development, and chronic low-grade inflammation, are believed to contribute to these reduced outcomes in obese women [9,10]. Understanding these mechanisms is vital for developing interventions that could enhance IVF success rates in this population.

Objectives

This analysis will aim to make systematic assessments of obesity’s impact on key IVF outcomes, such as implantation rates, CPRs, LBRs, and miscarriage rates. By summarizing the literature, the review hopes to give clinicians a sound, evidence-based understanding of the effects of obesity on IVF success, as well as therapeutic avenues to prevent them. These are the specific review objectives: (1) To evaluate the impact of obesity on IVF success rates and stages of the process. This objective focuses on how obesity affects CPRs, LBRs, and miscarriage rates while also examining the influence of obesity on different stages of IVF, including ovarian stimulation, follicle maturity, and embryo development. (2) To investigate biological mechanisms linking obesity and reproductive outcomes. This review will explore the hormonal imbalances, inflammation, and metabolic disruptions associated with obesity, which negatively affect reproductive health. (3) To highlight interventions to improve outcomes in obese patients. This objective aims to identify effective interventions, including weight loss strategies and treatment modifications, to enhance IVF outcomes for obese patients. (4) To identify gaps in the literature and suggest areas for future research. The review will highlight areas where further research is needed to improve understanding and care for obese individuals undergoing IVF, including the need for counseling for emotional well-being in these women.

## Review

Methods

Search Strategy

We did an extensive literature search in five large databases: PubMed/PubMed Central (PMC), Cochrane, Science Direct, MDPI, and PubMed Medical Subject Headings (MeSH) Library. We searched these databases between January 2023 and May 2024 to see if obesity was affecting outcomes in IVF. MeSH and free text were also used to complete the search. Keywords used included "Obesity", "In-Vitro Fertilization", OR "IVF", "ART", and "Pregnancy Rates". Filters were used to limit the search to research conducted with humans in English and peer-reviewed journals. Unpublished data, conference abstracts, and dissertations were not accepted as grey literature to preserve a rigorous evidence level. Table 1 shows the search strategy used for this article and the number of articles identified in each database. The inclusion and exclusion criteria used are shown in Table 2.

**Table 1:** Search strategy used for this article and number of articles identified in each database. MeSH: Medical Subject Headings; PMC: PubMed Central; IVF: in vitro fertilization.

Search strategy	Database used	Number of papers identified
Impact of obesity on IVF outcomes	PMC	127
Obesity and infertility or IVF treatment outcomes	Cochrane	75
Impact of obesity on IVF outcomes	Science Direct	177
Obesity and Infertility	MDPI	112
(Obesity AND ((ffrft[Filter]) AND (clinicaltrial[Filter] OR meta-analysis [Filter] OR randomizedcontrolledtrial[Filter] OR review [Filter] OR systematicreview[Filter]) AND (humans [Filter]) AND (female [Filter]) AND (english[Filter]) AND (2009:2024[pdat]))) AND (IVF fertilization AND ((ffrft[Filter]) AND (clinicaltrial[Filter] OR meta-analysis [Filter] OR randomizedcontrolledtrial[Filter] OR review [Filter] OR systematicreview[Filter]) AND (humans [Filter]) AND (female [Filter]) AND (english[Filter]))) Filters: Free full text, Clinical Trial, Meta-Analysis, Randomized Controlled Trial, Review, Systematic Review, Humans, English, Female	PubMed MESH	29
((Obesity AND ((ffrft[Filter]) AND (clinicaltrial[Filter] OR meta-analysis [Filter] OR randomizedcontrolledtrial[Filter] OR review[Filter] OR systematicreview[Filter]) AND (humans[Filter]) AND (female[Filter]) AND (english[Filter]) AND (2009:2024[pdat]))) AND (Infertility AND ((ffrft[Filter]) AND (clinicaltrial[Filter] OR meta-analysis[Filter] OR randomizedcontrolledtrial[Filter] OR review[Filter] OR systematicreview[Filter]) AND (humans[Filter]) AND (female[Filter]) AND (english[Filter])))) AND (IVF outcomes AND ((ffrft[Filter]) AND (clinicaltrial[Filter] OR meta-analysis[Filter] OR randomizedcontrolledtrial[Filter] OR review[Filter] OR systematicreview[Filter]) AND (humans[Filter]) AND (female[Filter]) AND (english[Filter]))) Filters: Free full text, Clinical Trial, Meta-Analysis, Randomized Controlled Trial, Review, Systematic Review, Humans, English, Female	PubMed MESH-included IVF articles	13
Total number of articles identified		533
Number of articles remaining after removing duplicates		323

**Table 2:** Inclusion and exclusion criteria. IVF: in vitro fertilization.

Inclusion criteria	Exclusion criteria
Published in peer-reviewed journals	Grey literature (unpublished data, conference abstracts, dissertations), not published in peer-reviewed journals
Studies reporting outcomes of IVF treatment (implantation rates, clinical pregnancy rates, live birth rates, miscarriage rates)	Studies not focusing on IVF outcomes
Humans undergoing IVF treatment	Animal studies
English language studies	Studies in languages other than English
Women with obesity (BMI ≥ 30 kg/m²) undergoing IVF	Studies without a clear definition of obesity (BMI ≥ 30 kg/m²)
Full free-text articles with studies in adults	Studies focusing on pediatric populations

The Preferred Reporting Items for Systematic Reviews and Meta-Analyses (PRISMA) 2020 standards were followed in this systematic review.

Screening Process and Study Selection

All the collected articles were moved to EndNote (Clarivate, Philadelphia, PA), and duplicate articles were removed. The team screened the articles first through their titles and abstracts. Further evaluation was conducted by reviewing the full text of the shortlisted articles to ensure relevance. The inclusion and exclusion criteria were applied, and only the articles that fulfilled all requirements were selected for the review. Throughout the process, all team members contributed equally. Titles and abstracts were independently screened by the team, and discrepancies were resolved through team discussions and consensus. Key data points, including study design, population characteristics, and IVF outcomes, were independently extracted by each member.

Quality Assessment

The studies in this review were rated according to the study design, with appropriate quality assessment tools. We used the Newcastle-Ottawa Scale (NOS) to score cohort studies for selection, comparability, and outcomes. The included randomized controlled trials (RCTs) were reviewed using the Cochrane Risk of Bias Tool, and three main areas were looked at, including randomization, blinding, and reporting. Systematic reviews were tested for comprehensiveness and methodological strictness using the Scale for the Assessment of Narrative Review Articles (SANRA) checklist, and the case report was evaluated for clinical validity and transparency according to CARE (CAse REports) guidelines.

Overall, the cohort studies demonstrated moderate to high quality, though many lacked sufficient control for confounding factors. The RCT had a moderate risk of bias due to insufficient blinding despite a strong randomization process. Systematic reviews were generally well-conducted, and the case report was of good quality based on a retrospective analysis of seven women with polycystic ovary syndrome (PCOS) undergoing IVF treatment.

Data Extraction and Synthesis

To get relevant data, the study design, sample size, population demographics, BMI categories, IVF technique (fresh or frozen transfer), and IVF outcome (clinical pregnancy rates, live birth rates, miscarriage rates, embryo quality) were gathered through a systematic data extraction procedure. Since studies vary so much in design, outcome, and BMI limits, we did a narrative synthesis. We ran subgroup analyses by BMI categories and IVF regimens where relevant. Heterogeneity was assessed qualitatively, and confounding factors such as age and ovarian reserve were taken into account. No statistical analysis was performed in writing this review. Only the analysis performed by the referencing articles was stated to analyze and compare with similar studies. Appropriate citation was used wherever required.

Results

**Figure 1:**
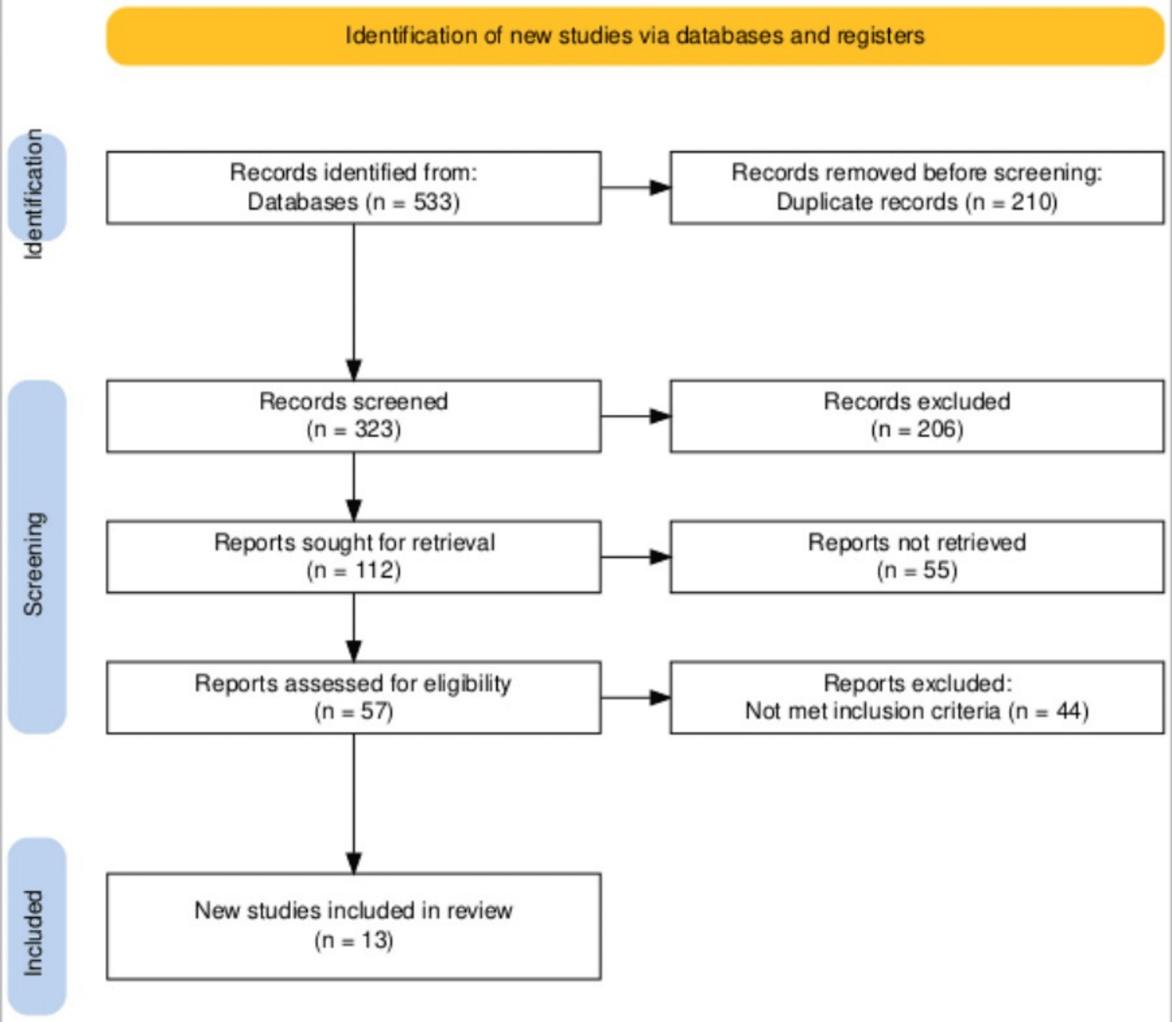
Preferred Reporting Items for Systematic Reviews and Meta-Analyses (PRISMA) flowchart. Haddaway et al. [11].

A total of 13 studies were included in the review, comprising six retrospective cohort studies, one RCT, two systematic reviews, three narrative reviews, and one case report. These studies investigated the effects of obesity on IVF outcomes, with a focus on clinical pregnancy rates, live birth rates, miscarriage rates, and the underlying biological mechanisms that explain these associations. Obese women were defined according to the World Health Organization (WHO) classification as normal weight (18.5-24.9 kg/m^2^), overweight (25-29.9 kg/m^2^), and obese (>30 kg/m^2^). Figure 1 shows the PRISMA flowchart created to represent the number of articles at each stage [1-22].

Quality Check

Cochrane risk of bias assessment for RCTs (Table 3), NOS for cohort studies (Table 4), and SANRA checklist for systematic reviews were used (Table 5).

**Table 3:** Cochrane risk of bias assessment table for the single randomized controlled trial.

Study	Selection bias	Allocation concealment	Performance bias	Detection bias	Attrition bias	Reporting bias	Overall risk of bias
Svensson et al. (2022) [10]	Low risk	Low risk	Unclear risk	Unclear risk	Low risk	Low risk	Moderate risk

**Table 4:** Newcastle-Ottawa Scale (NOS) quality assessment of observational studies.

Authors/year of publication	Selection	Comparability	Outcome
Zeng et al. (2023) [1]	***	*	***
Vural et al. (2015) [4]	***	**	***
Lin et al. (2024) [5]	***	*	***
Brunet et al. (2020) [12]	***	**	***
Liu et al. (2023) [18]	***	*	**
Ozekinci et al. (2015) [21]	***	**	**

**Table 5:** SANRA checklist for systematic reviews. SANRA: Scale for the Assessment of Narrative Review Articles.

Study	Justification of the article's importance for the readership	Statement of concrete aims or formulation of questions	Description of the literature search	Referencing	Scientific reasoning	Appropriate presentation of data	Total score
Jeong et al. (2024) [3]	2	2	1	2	2	2	11
Jungheim et al. (2013) [19]	2	2	2	2	2	2	12

Study Characteristics

This systematic review analyzed 13 studies on obesity's effects on outcomes in IVF. These studies have sample sizes ranging from small groups of 25 patients to large observational studies with 44,773 subjects. All of these were conducted on women who received IVF treatments; some were done specifically with overweight and obese women (BMI ≥ 30 kg/m²), and one also included male BMI as a predictor of IVF success. The outcomes of IVF treatment were recorded from all the included studies. Interventions compared were weight-loss interventions, IVF methods, and medication co-treatments across the BMI categories. These research studies also give us a more specific picture of how obesity, especially in women, affects IVF success, oocyte quality, embryo quality, and pregnancy. Below is a table showing the summary of the included studies (Table 6).

**Table 6:** Characteristics of included studies. RCT: randomized controlled trial; IVF: in vitro fertilization; ART: assisted reproductive technology; ICSI: intracytoplasmic sperm injection; PCOS: polycystic ovary syndrome; GnRH: gonadotropin-releasing hormone; CPR: clinical pregnancy rate; LBR: live birth rate; LH: luteinizing hormone; hsCRP: high-sensitivity C-reactive protein.

Author/year	Study design	Sample size	Population	Interventions/comparisons	Outcomes measured	Key findings
Zeng et al. (2023) [1]	Retrospective cohort study	44,773 women	Women undergoing fresh/frozen IVF cycles, grouped by BMI (<18.5, 18.5-23.9, 24.0-27.9, ≥28.0 kg/m²)	Comparison of clinical pregnancy, live birth, and miscarriage rates in different BMI groups	Clinical pregnancy rate, live birth rate, and miscarriage rate	Obesity was associated with increased miscarriage rate but did not affect live birth rates. Overweight and obese women showed higher clinical pregnancy rates in frozen-thawed cycles.
Elnashar (2023) [2]	Narrative review	N/A	Women with obesity undergoing IVF	Obesity and its effects on assisted reproductive technology	Obesity leads to decreased CPR, LBR, and increased miscarriage rates	Obesity significantly impacts IVF success, affecting oocyte and endometrial function
Jeong et al. (2024) [3]	Systematic review and meta-analysis	1627 women	Overweight and obese women undergoing IVF (BMI ≥ 25 kg/m²)	Weight loss through lifestyle modification or medication vs. no weight loss	Live birth rate, clinical pregnancy rate, and miscarriage rate	Weight loss before IVF did not significantly improve live birth, pregnancy, or miscarriage rates
Vural et al. (2015) [4]	Retrospective cohort study	188 women	Poor ovarian responders (BMI categories: normal, overweight, obese)	Comparison of IVF outcomes in different BMI groups of poor ovarian responders	Clinical pregnancy rate, fertilization rate, and LH levels	Obese women had significantly lower fertilization and pregnancy rates despite similar oocyte count; LH levels were lower in obese women
Lin et al. (2024) [5]	Retrospective cohort	704 women	Overweight women undergoing IVF with or without letrozole co-treatment	Letrozole co-treatment vs. standard GnRH antagonist protocol	Live birth rate, clinical pregnancy rate, and miscarriage rate	Letrozole co-treatment was associated with a significantly higher live birth rate (38.7% vs. 22.6%) and no significant differences in miscarriage rate or neonatal outcomes
Purcell & Moley (2011) [7]	Narrative review	N/A	Women with obesity undergoing ART	Impact of obesity on oocyte quality and development	Obesity negatively affects oocyte maturation and metabolism	Obesity impairs oocyte quality, leading to subfertility
Liu et al. (2023) [18]	Retrospective cohort	3874 women	Women and men undergoing IVF, grouped by BMI (normal, overweight, obese)	Impact of male and female BMI on IVF outcomes	Fertilization rate, embryo quality, and live birth rate	Female obesity is associated with reduced fertilization rate and embryo quality, while male BMI has a smaller effect on outcomes
Gautam et al. (2023) [9]	Narrative review	N/A	Women with obesity undergoing ART	Comparison of fertility outcomes in obese vs. non-obese women	Impact of obesity on fertility and IVF outcomes	Obesity impairs oocyte quality, endometrial receptivity, and pregnancy rates
Svensson et al. (2022) [10]	Prospective multicenter RCT	195 (128 control, 59 intervention)	Overweight and obese women undergoing IVF (BMI ≥ 30 and < 35 kg/m²)	The control group received IVF directly, and the intervention group underwent a weight reduction program before IVF	Live birth rate and pregnancy rate	Weight reduction did not improve live birth or pregnancy rates; inflammation markers (hsCRP, leptin) were not predictive of outcomes
Brunet et al. (2020) [12]	Retrospective cohort	1588 women	Women undergoing IVF (BMI categories: normal, overweight, obese)	Comparison of live birth rate (LBR) across BMI categories	Live birth rate (LBR) and miscarriage rate	No significant impact of obesity on cumulative LBR; miscarriage rates were unaffected by obesity severity
Jungheim et al. (2013) [19]	Systematic review and meta-analysis	4758 women	Women undergoing IVF with donor oocytes (BMI ≥ 30 kg/m²)	Obesity vs. normal BMI in IVF using donor oocytes	Implantation rates, clinical pregnancy rates, miscarriage, and live birth rates	Obesity did not significantly impact clinical pregnancy or live birth rates in donor oocyte recipients
Ozekinci et al. (2015) [21]	Retrospective cohort	298 women	Women undergoing their first IVF-ICSI cycles (BMI categories: normal, overweight, obese)	IVF outcomes compared across BMI categories	Gonadotropin dose, stimulation duration, and pregnancy rates	Obese women required significantly higher gonadotropin doses and longer stimulation duration, but pregnancy outcomes were similar across BMI categories
Silvestrim et al. (2019) [22]	Case report	7 patients	Overweight/obese women with PCOS undergoing ART	Impact of lifestyle changes on ART outcomes	Fertilization rate, embryo quality, live birth	3 out of 7 women had successful gestations with lifestyle changes

The studies analyzed in this review showed that obesity had a negative impact on IVF success, as explained through various measures of IVF failure.

Clinical Pregnancy Rate and Live Birth Rate

Retrospective cohort studies by Brunet et al. (2020), Vural et al. (2015), and Lin et al. (2024) showed a 10-15% decrease in clinical pregnancy rates for obese women [4,8,12]. This decline was further supported by a narrative review done by Elnashar (2023), which reported a 7% decrease in live birth rates for every five-unit increase in BMI (RR: 0.85) [2]. In another study, involving 1144 infertile women treated by artificial donor insemination, women with a BMI of 28-36 had a relative risk of unsuccessful ovulation induction of 2.7 (95% CI =2.1-3.4) compared with women of lower or normal body weight (BMI: 20-24) [13]. Additionally, the prospective multicenter RCT done by Svensson et al. (2022) found significantly lower implantation rates (24% vs. 35%, p < 0.05) and higher miscarriage rates in obese women undergoing IVF compared to their non-obese counterparts [10].

Miscarriage Rate

Obesity was also linked to higher miscarriage rates across the studies. A retrospective cohort study done by Lo et al. (2012) demonstrated a 20-30% increase in miscarriage rates in obese women in couples with unexplained recurrent miscarriage (OR: 1.73, 95% CI: 1.06-2.830), while another retrospective study done by Zeng et al. confirmed that obese women were 1.7 times more likely to experience first-trimester miscarriages, particularly in frozen-thawed embryo transfers (adjusted OR = 1.42, 95% CI: 1.05-1.92) [1,14].

Chromosomal Abnormality Increase

Obesity was associated with decreased IVF success, as it contributed to lower fertilization rates and a higher incidence of chromosomal abnormalities in embryos, underscoring the negative influence of high BMI on reproductive outcomes. A retrospective cohort study done by Lin et al. shows that letrozole co-treatment in overweight women undergoing IVF resulted in a significantly higher live birth rate of 38.7% compared to 22.6% in the antagonist-only group (P = 0.026), with an adjusted odds ratio (OR) of 2.00 (95% CI: 1.17-3.39, P = 0.011), highlighting its potential benefit in improving IVF outcomes [5].

Although the total number of follicles decreased and the retrieval of oocytes was reduced in the letrozole group, the rate of usable embryos was much higher (82%) than in the control (70%) (P = 0.002), suggesting an improvement in the embryo quality [5]. Such observations were also bolstered by a systematic review and meta-analysis of the literature by Jeong et al. (2024) that obesity severely reduces live births after IVF [3]. A live birth rate that is about 15% lower in obese women compared with their healthy-weight peers, with a risk ratio of 0.85 (95% CI: 0.82-0.87), highlights obesity's adverse effect on successful pregnancies after IVF [3].

Endometrial Receptivity

Endometrial receptivity, which plays a critical role in embryo implantation, was also compromised in obese women. A clinical trial done by Bellver et al. (2021) demonstrated that hormonal imbalances and inflammatory markers in the endometrium reduced receptivity, leading to lower implantation rates in obese women. One-day displacement of the window of implantation (dWOI) was directly associated with high BMI, and significant differences were found between the non-obese and obese categories (9.7% vs. 25.3 %, respectively; p = 0.02), with the highest degree of dWOI in patients with class II-III obesity [15]. Another systematic review and meta-analysis done by Jeong et al. (2024) suggests that in obese women using their own oocytes, poor embryo quality has been linked to reduced IVF success. However, in donor oocyte recipients, where the oocytes come from young, healthy donors, the primary concern shifts to endometrial receptivity rather than embryo quality itself. This distinction implies that the negative effects of obesity on embryo quality may be more relevant in autologous IVF (using one’s own oocytes) rather than in donor oocyte cycles [3].

Metabolic Disturbances and Maternal Complications

The biological mechanisms linking obesity to poorer IVF outcomes were explored in several studies. A narrative review article by Purcell and Moley (2011) highlighted the role of hormonal imbalances, such as increased insulin resistance and elevated androgen levels, in disrupting ovarian function and reducing oocyte quality [7]. In addition, a prospective multicentric RCT conducted by Svensson et al. (2022) identified chronic low-grade inflammation and oxidative stress in obese women as key factors contributing to reduced embryo quality and impaired endometrial receptivity [10]. A longitudinal cohort study by Luke et al. (2017) further supported this by reporting elevated levels of inflammatory markers, such as tumor necrosis factor-alpha (TNF-α) and IL-6, which were associated with reduced implantation and clinical pregnancy rates [16].

Li et al. (2023) reported in their prospective cohort study that, in women who conceived with IVF, obesity was a risk factor for gestational diabetes, pre-eclampsia, and other maternal complications. These results point to the metabolic and inflammatory alterations that obesity brings not only to IVF success but to pregnancy complications as well. This reflects the need for preconception counseling and specific interventions for obese women on fertility treatment to enhance IVF results and maternal health [17].

Liu et al. (2022), in a cohort study of 11,191 IVF couples, studied male and female overweight/obesity and their impact on IVF. Their research showed that female overweight/obesity and combined male/female overweight/obesity in the initial fresh IVF-embryo transfer cycles predicted significantly fewer available and good-quality embryos (p < 0.05). The fertility rate (p < 0.001) and the average fertilization rate (p < 0.001) were also significantly lower in the female overweight/obesity group. The male/female overweight/obesity group as a whole had marked declines in the numbers of available embryos (p = 0.002), quality embryos (p = 0.010), and rates of fertilization (p = 0.001) [18].

Need for Further Research

Jungheim et al. (2013), in a systematic review and meta-analysis of the effect of obesity on IVF results in donor oocyte recipients, evaluated 4758 women. They did not report an association between obesity (BMI ≥ 30 kg/m²) and success in pregnancy; the RR for pregnancy was 0.98 (95% CI: 0.83-1.15), with no effect on implantation rate (RR: 0.93, 95% CI: 0.80-1.07), miscarriage (RR: 1.12, 95% CI: 0.83-1.50), or live birth (RR: 0.91, 95% CI: 0.65-1.27). This means that in donor oocyte cycles, endometrial receptivity for obese women might not be as important as oocyte quality (as in autologous oocyte IVF cycles). This makes it imperative that more research is needed to establish the influence of obesity on IVF success in terms of oocyte type and quality [19].

**Figure 2:**
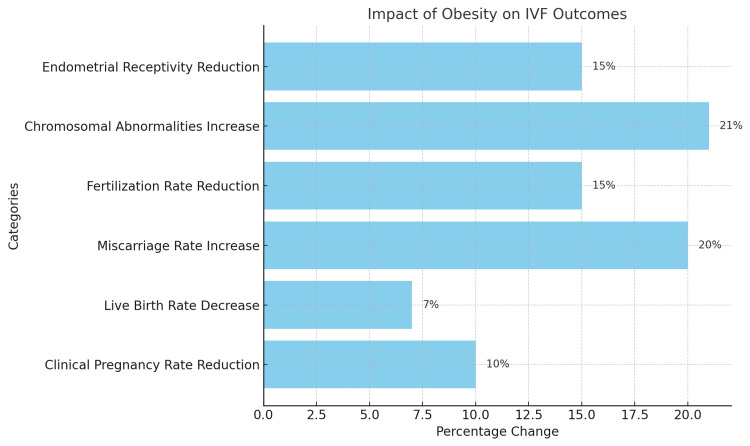
Graphical representation of the findings from the included studies. Graph created by Dr. Sruthi Boddeti. IVF: in vitro fertilization.

Below is a graphical representation of the findings, showing the percentage changes in various outcomes related to the impact of obesity on IVF success rates (Figure 2). This bar chart highlights reductions in clinical pregnancy rates, live birth rates, fertilization rates, and endometrial receptivity, along with increases in miscarriage rates and chromosomal abnormalities.

Discussion

Comparison With Previous Research

This review is in agreement with most of the research already showing that obesity has a detrimental effect on IVF. Several large studies (one meta-analysis included more than 900,000 cycles of IVF) have demonstrated that CPR and LBR significantly decrease as BMI rises [2,20]. The 5-7% decline in LBR with each five-unit increase in BMI is consistent with other reports of obesity-related decreases in IVF success compared with normal-weight women [2]. Additionally, Ozekinci et al., in a retrospective cohort study of 298 IVF-intracytoplasmic sperm injection (ICSI) cycles, found that although obese women required significantly higher gonadotropin doses (p < 0.001) and longer stimulation durations (p = 0.008), BMI did not notably impact the number of retrieved oocytes, fertilization rates, or pregnancy outcomes (p > 0.05). These findings highlight how obesity can influence hormonal requirements in IVF-ICSI cycles [21].

Similarly, the higher miscarriage rates observed in this review have been corroborated by numerous studies, particularly in frozen-thawed blastocyst transfers [1]. The relationship between obesity and increased miscarriage rates, largely driven by metabolic and endocrine disruptions, is well-documented [7,22]. These findings further emphasize the need for weight management interventions prior to IVF to mitigate such risks.

However, while the impact of obesity on oocyte and embryo quality is well-documented in this review, the literature differs a bit on the magnitude of this impact. There is also some research suggesting that BMI has no direct influence on the quality of oocytes and embryos, unlike implantation rates or pregnancy outcomes. This discrepancy shows how obesity alters reproduction at different stages and that there is still a lot to be discovered.

Mechanisms of Impact

The adverse IVF outcomes caused by obesity have several biological mechanisms at work. Hormonal dysfunctions such as insulin resistance and hyperandrogenism halt normal ovarian function and depress oocyte maturation [10,22]. This hormonal dysregulation also affects the endometrial environment, reducing implantation rates. It has been reported through genome analyses that there are alterations in the gene expression of the endometrium in obese women, further diminishing endometrial receptivity [2,3,7].

Chronic low-grade inflammation and oxidative stress are other major pathways that influence IVF results. Higher levels of pro-inflammatory mediators like tumor necrosis factor and IL-6 have been linked to lower-quality embryos and lower rates of implantation in obese women [10].

**Figure 3:**
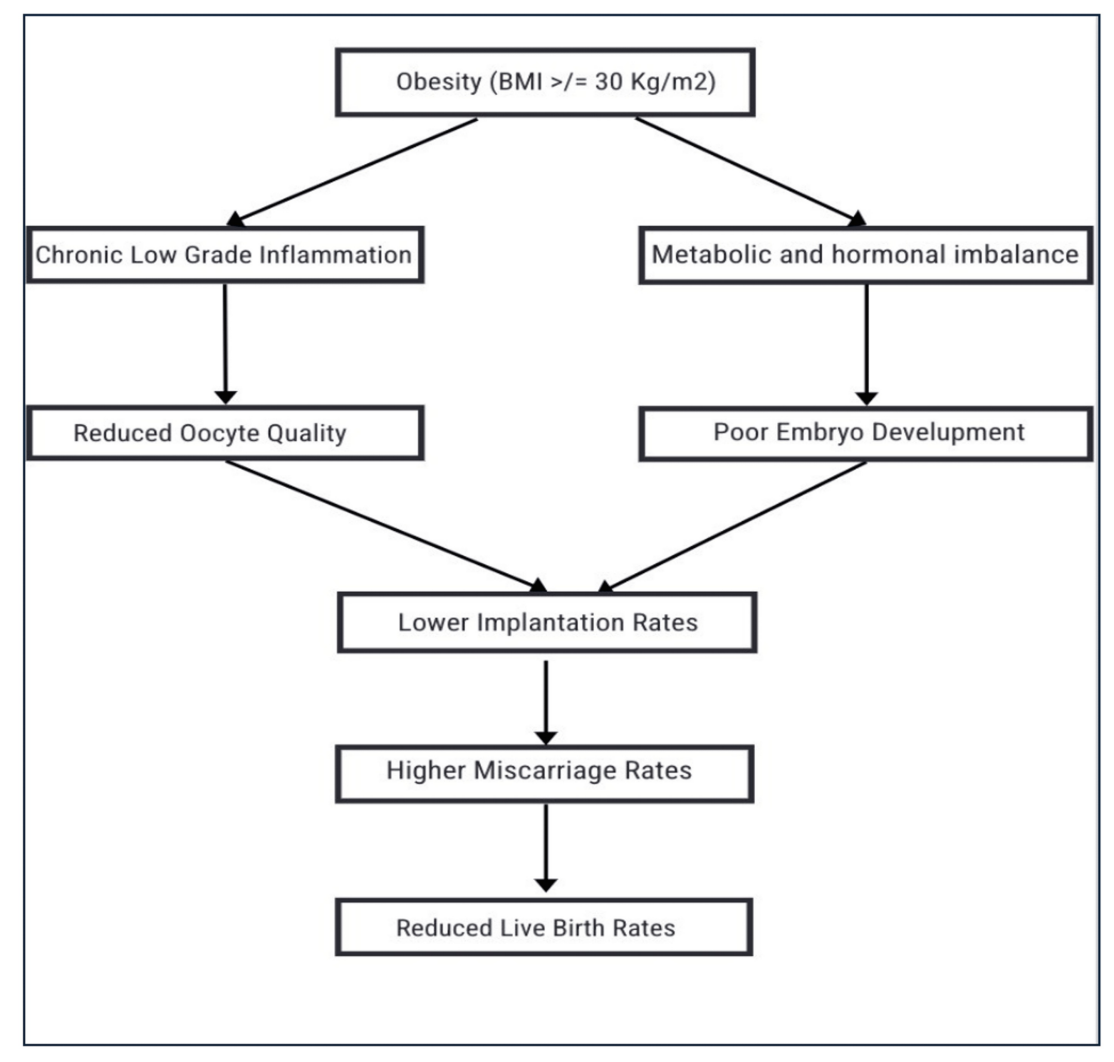
Flowchart showing various mechanisms by which obesity is impacting fertility. Flowchart created by Dr. Sruthi Boddeti.

Further, metabolic dysfunctions, especially those related to lipid metabolism, can contribute to low-quality oocytes and malformed embryos. This increased frequency of chromosomal defects in embryos from obese and elderly patients suggests that metabolic errors are responsible for decreased embryo viability (Figure 3) [10,16,22].

Clinical Implications

What this review highlights for clinical practice has direct implications for the treatment of obese women undergoing IVF. Given that obesity is clearly related to poorer IVF success, weight management should be an integral part of pre-IVF care. The effects of even a slight weight loss (5-10%) have been reported to boost fertility by balancing hormones, increasing oocyte quality, and decreasing miscarriage rates [9,15].

In clinical settings, optimizing IVF protocols for obese patients could help mitigate some of the adverse effects. Adjusting ovarian stimulation protocols, as suggested by one of the studies, may increase clinical pregnancy rates, although it may not entirely counteract the reduced embryo quality associated with obesity [1,5]. Additionally, providing psychological support for obese women during the IVF process is essential, as they often face additional stress and anxiety due to body image issues and societal stigma [9].

Counseling patients on the importance of weight management and addressing potential psychological challenges can improve both physical and emotional outcomes, making IVF treatment more holistic and patient-centered.

Strengths

This review has thoroughly explored the varying conclusions of relevant literature. We had been successful in identifying interventions that can reduce IVF failures in obese women. Gautam et al. (2023) emphasized the benefits of pre-IVF weight loss interventions, including lifestyle modifications and bariatric surgery, associated with improved IVF success rates [9]. These benefits were most pronounced when weight loss occurred six to 12 months prior to IVF treatment. The prospective multicenter RCT by Svensson et al. (2022) reported that optimizing ovarian stimulation protocols in obese women resulted in a modest increase in clinical pregnancy rates (38% vs. 34%, p < 0.05). However, this intervention did not fully mitigate the negative effects of obesity on embryo quality [10]. This review only included the literature most relevant to the question about the effects of obesity on IVF outcomes; hence, it would provide a single platform for clinicians and researchers to better understand and implement the interventions.

Limitations

There are some limitations to the studies included in this review. First, a few of the retrospective cohort studies lacked adequate control for confounding factors such as age, ovarian reserve, and underlying health conditions, which could have influenced the outcomes. Furthermore, there is variability in BMI classification and IVF protocols from study to study, which can make findings less generalizable.

Another constraint is the low number of RCTs available for inclusion in the review. Cohort studies can be very useful for observational data, but more RCTs would add to the evidence, particularly in comparison of the effectiveness of treatments for better IVF outcomes in obese patients.

Future Research Directions

Future studies should look more systematically to see whether weight management interventions, including lifestyle changes and bariatric surgery, improve IVF results in obese women. If we could discover when weight loss would be most beneficial relative to IVF, that would be of great help to physicians [3]. Moreover, we still need more research to describe the exact molecular mechanisms that correlate with obesity and IVF failure. The detection of specific inflammatory markers, hormones, and metabolic dysregulation may be able to translate into targeted treatments to improve the reproductive outcomes in obese women [2]. Likewise, longitudinal studies that study the long-term health outcomes of children born to overweight mothers with IVF would be helpful. This would help determine whether maternal obesity has lasting effects on offspring beyond pregnancy and birth outcomes [23].

Obesity’s emotional and psychological impact on patients undergoing IVF was also discussed in several studies. A case report described the emotional challenges faced by an obese patient undergoing IVF, highlighting the need for psychological support throughout the process [22]. One of the narrative review articles stressed that obese women may face heightened levels of stress and anxiety due to societal stigmatization, body image concerns, and repeated IVF failures, underscoring the importance of comprehensive emotional and psychological care [9]. This shows that more studies should explore the psychological and emotional impact of obesity on IVF success, as well as the effectiveness of mental health interventions in improving the IVF journey for obese patients.

Recommendations

Weight Management as a Preconception Strategy

Weight management should be a top priority for obese women planning to have IVF. Researchers have shown that even a small weight loss (5-10%) before starting an IVF cycle can dramatically increase reproductive success by restoring hormonal balance, boosting oocyte quality, and decreasing miscarriage. In obese patients, lifestyle advice, including diet and exercise programs, should take center stage in clinicians' preconception care. Bariatric surgery can also be pursued for very obese women, as sustained weight loss is associated with improved IVF success.

Tailored IVF Protocols

Altering the protocol for ovarian stimulation and choosing the right fertility drugs according to BMI can help. Also, careful assessment of embryo quality and tailored embryo transfer protocols according to the patient's individual metabolic characteristics can counteract the detrimental effects of obesity. More studies will need to focus on building and perfecting these custom protocols to make IVF more effective for obese patients.

Addressing Comorbidities

For obese women, there is a risk of comorbid conditions, including PCOS, insulin resistance, and type 2 diabetes, that can make IVF less successful. Treating these comorbidities in tandem with infertility treatments is important to ensure reproductive success. Practitioners need to take an integrated multidisciplinary approach, including endocrinologists, dietitians, and reproductive specialists, to address these conditions as a whole. With appropriate control of comorbidities, the negative impact of obesity on IVF might be minimized.

Personalized and Multidisciplinary Care

Obese women receiving IVF require more individualized and multidisciplinary care. It is not just about adapting to the IVF regimens but also the psychological and emotional difficulties that obesity brings. Counseling and psychotherapy are vital in supporting patients with stress and anxiety around infertility and body image. When we combine dietitians, mental health workers, and reproductive endocrinologists in a multidisciplinary care team, all three dimensions of the patient's health (physical, emotional, and psychological) are covered. This integrated care can enhance the reproduction as well as the well-being of this particular cohort of patients.

## Conclusions

In summary, obesity adversely affects IVF outcomes with a reduction in oocyte quality, lower embryo development, endometrial receptivity, and higher miscarriages. Compared with women of normal weight, women with BMI ≥ 30 kg/m² who receive IVF treatment have significantly lower CPR and LBR. These detrimental effects are multifactorial and are associated with a complex interplay of hormonal disturbances, metabolic disorders, and chronic low-grade inflammation. Given the growing scientific evidence that obesity causes poor IVF outcomes, weight-related interventions, optimized IVF protocols, and comprehensive patient care are necessary to improve reproductive success in this population. These findings underscore the need to include weight management and lifestyle modification in fertility counseling and also highlight the importance of broader public health measures to prevent obesity and improve reproductive outcomes at a population level.
